# Supplementary material for: Riddles in the cold: Antarctic endemism and microbial succession impact methane cycling in the Southern Ocean
Source: Proc Biol Sci. 2020 Jul 22;287(1931):20201134. doi: 10.1098/rspb.2020.1134 (PMC7423672; doi:10.1098/rspb.2020.1134)
Supplement: Supplemental Results, Discussion, Figures and Tables, and Data Analysis Pipeline. [file rspb20201134supp1.docx]

**Supplemental Materials for :**

Thurber AR, Seabrook SA, Welsh RM. 2020. Riddles in the cold: Antarctic endemism and microbial succession impact methane cycling in the Southern Ocean. *Proceedings of the Royal Society B: Biological Sciences*. 20201134. <http://dx.doi.org/10.1098/rspb.2020.1134>

**Supplementary Results and Discussion:**

*Microbial Community Structure*

The sediment composition of the microbial community differed across years and site. Sequencing of 29 cores across 7 sites, two years and as many as 12 vertical fractions per core, resulted in 199 samples of which 3.9 million sequences were generated. After quality control this resulted in a mean sequencing depth of 16,047 ± 1,353 reads per sample (Supplemental Table 3) and we eliminated samples with fewer than 1,900 reads based on divergent community structure below this cut off as visualized through exploratory multidimensional scaling. This resulted in 12,979 unique Amplicon Sequence Variants (ASVs) with an average of 1,279±68 ASV per sample. At all habitats (Control, Reference, and the Cinder Cones Seep (CCS) in 2012 and 2016), ASV richness was lowest at the surface and plateaued at between 1-3 cm sediment depth (Supplemental Figure 3A). At the control sites, the ASVs identified fell mostly within two members of the Bacteroidetes phylum, the Flavobacteriales and Cellvibrionales (Figure 4). The proportion of ASVs from these two taxa decreased with increasing sediment depth, with relative abundance going from 11.2 ± 0.7% to 2.2 ± 0.5% and 11.3 ± 1.9% to 3.1 ± 0.7%, respectively. With increasing sediment depth ASVs identified as Desulfobacteriales increased from 1.2 ± 0.4% of the community to 9.2 ± 3.1% (Figure 4). In many ways, the microbial community at the Reference site was similar to the controls with the addition of ASVs belonging to the Camplylobacteriales which made of 3.8 ± 8% of the total ASVs; Camplylobacteriales ASVs only made up 0.15 ± 0.06% of the ASVs at the control sites.

The ASV composition at the CCS was dominated by taxa known to be sulfide oxidizers in both years, although there was a decrease in 2016 in comparison to 2012. In 2012, 64 ± 9% of the ASVs were identified as *Sulfurovum* (the only member of the Sulfurovaceae family found) and an additional 1.5 ± 0.6% percent were made up of ASVs identified as belonging within the Thiotrichaceae (Supplemental Figure 3B and 3C). Thiotrichaceae include the most common mat forming taxa at seeps, although the vast majority of taxa could not be identified below the family level in this group and they increased in relative proportion with depth which is not expected if they were the dominant mat forming taxa. In 2016, the mean percent of the microbial ASVs identified as *Sulfurovum* decreased to 24.1 ± 5.8% but *Thiotrochales* had increased to 2.5 ± 0.3%. While there were 21 ASVs within the *Sulfurovum* genus, 56% of the diversity was made up of a single ASV, an ASV that was present through both years. At the CCS, there were 77 ASVs within the Thiotrichaceae but 51% fell within a single ASV. ASVs of putative sulfide-oxidizing taxa made up a larger proportion of the total ASVs at the CCS in comparison to the Reference site where the percent of ASVs that fell within the *Sulfurovum* and *Thiotrochales* made up 1.5% and 2.2% of the total ASVs*,* respectively. However, even these abundances were driven almost entirely by a single Reference sample, the sample is clear in Figure 3 as it groups with the CCS sites. If we omit this one sample, then the mean percent of ASVs at the Reference site within the *Sulfurovum* and *Thiotrochales* was 0.05 ± .01 and 1.2% ± 0.1%. While this may suggest either a mislabeling or contamination of the one sample during processing, it is notable that the dominant *Sulfurovum* ASV in the CCS feature is a different ASV than the dominant one in the Reference sample. Further, if we look at the overall community composition of all of the samples (Figure 3B) we can see that there is a good agreement between the replicates of the Reference samples, especially with increasing depth. We instead interpret this to mean that the near references are episodically influenced by the seepage however with reduced fluid flow or, due to the high permeability of the sediments, that there is rapid exchange of the fluid throughout the neighboring sediment.

An integral part of seep ecosystems are sulfate reducing bacteria (SRB), which occur as members of syntrophic consortia with ANME, as well as outside of this symbiotic relationship. Globally, a diversity of Deltaproteobacteria are involved in this process including members of the *Desulfobacteracea* and *Desulfobulbaceae* that are further differentiated into SEEP SRB1-4 (summarized in [[1]](https://paperpile.com/c/drusII/3ssX9)). Seep SRBs were a common component of the CCS, with a total of 51 ASVs identified including groups of the SEEP1, SEEP2, and SEEP4 groups represented. These taxa were not limited to the CCS or the Reference site but were also present in the Control sites (Supplemental Figure 3D). When we compared SRB sequence to a global compilation of SRBs from seep sites (Supplemental Figure 4), these taxa form novel clades as well as clades inclusive of methane seep SRB including the sequence from the most proximate known methane seeps off of New Zealand as well as Arctic methane seeps.

Bacterial methane oxidation can be carried out by a diversity of phyla, however only the *Methylococcaceae* appeared to be involved in aerobic methane oxidation at the CCS. Other taxa known to be able to oxidize methane aerobically include the *Methylocystaceae (*Alphaproteobacteria ) *and Methylocella (*Alphaproteobacteria)[[2,3]](https://paperpile.com/c/drusII/9k3s+qZlT) and there is evidence that both *Methylophilaceae (Betaproteobacteria)* and *Methylophaga (*Gammaproteobacteria) are also potentially capable of methane oxidation [[4]](https://paperpile.com/c/drusII/WzuY). Methane oxidation is also known from the widespread phylum *Verrucomicrobia* [[5]](https://paperpile.com/c/drusII/ME05) however the methanotrophic members of this phylum are thought to be exclusively mesophilic or thermoacidophilic [[2,6,7]](https://paperpile.com/c/drusII/TGMr+9k3s+31pJ). Of all of these groups, ASVs were only found at the CCS that were identified as *Methylococcaceae* and members of the *Verrucomicrobia*. While the Verrucomicrobia were present, and in certain instances ASVs belonging to this group made up more than 10% of the microbial ASVs recovered (Supplemental Figure 3E), they made up the greatest proportion of the ASVs at the control sites making it unlikely that they are active methane oxidizers in this habitat.

*Archaeal Community Composition*

Archaea in the CCS had a greater diversity at the Order level than at the Control Sites (Supplemental Figure 3F). Within the Archaea, ASVs that fell within the Woesearchaeia (Figure 4) made up the largest percentage of the Archaeal community across all sites below the surface 2cm of Sediment. The most abundant were a group of Woesearchaeia were not identifiable below Class. ASVs from this group increased with depth from 0.3±0.1% to >6±1% of the total microbial community at depths between 7 and 10cm, averaged across all sites. Other members of the Woesearchaeia were also common, for example the GW2011_AR13 group (Figure 4). Woesearchaeia are most likely anaerobic heterotrophs with genomic modeling approaches suggesting they may occur in a syntrophic partnership with methanogens [[8]](https://paperpile.com/c/drusII/sIzLV) however their occurrence across all of our sites, including the Controls, would make their role in methanogensis unexpected based on their habitat distributions.

The other Archaeal taxa with both abundant and common ASVs across all sites were the Nitrosopumilales (Figure 4). This group was most abundant at the surface and decreased in relative proportion with depth, however they were much more dominant within the control samples compared to the seep samples, making up as much as 3.8+0.4% of the microbial community at depth in those samples. In contrast they made up less than 1% of the community within all depths of the seep sites. This group is known to contain ammonia oxidizing chemolithoautotrophs[[9]](https://paperpile.com/c/drusII/NB8qY) and may provide non-seep fueled chemosynthesis at all sites sampled including the control areas.

Methane cycling was present as ASVs belonging to methanogens across two phyla and four Orders including the Methanosarcinales, the Methanofastidiosales, and the Methanomassiliicoccales and the Bathyarchaeia. ASVs from the best known of these groups, the Methanosarcinales, was largely absent from the control sites, never exceeding 0.002% of the microbial community which was sharply contrasted by the CCS in 2016 where this group increased from 0.02±0.01% at the surface to varying between 0.08 to 0.23% of the microbial community. In 2012, Methanosarcinales ASVs were far more abundant reaching 1.0±0.2% of the community at 5-7cm sediment depth. ASVs belonging to the other taxa were not common members of the community, all having a max percentage of less than 0.1% with the exception of a single 8-9cm fraction within the 2016 seep that had 0.2% of the ASVs identified as Methanofastidiosales. Instead, what is remarkable is the diversity of methanogens at the sites. It is only in the past few years that the Methanofastidiosa and the Methanomassiliicoccales have been implicated in the cycling of methane. Further, the role of Bathyarchaea in general is still being uncovered, and they may be involved in methanotrophy [[10]](https://paperpile.com/c/drusII/5nLA9). Further, across this group of methanogens a wide variety of methanogenic pathways are implicated [[11]](https://paperpile.com/c/drusII/L7FL2) suggesting the CCS may be an intriguing place to understand why there is such high methanogenic diversity.

The other Archaeal group that were represented based on ASVs were members of the Asgard group, including Heimdallarchaeia, Lokiarchaeia,and Odinarchaeia, in addition to a further group that was unresolved below the phylum level. ASVs from these taxa were not abundant, never more than 1 percent of the community (Supplemental figure 3H), however the potential role of Lokiarchaea as the ancestral eukaryotes [[12]](https://paperpile.com/c/drusII/YjGQP), the high diversity Asgardian Orders at the CCS, and our understanding of Antarctic endemism from polar metazoans (see main text), makes this discovery provocative.

Overall the microbial community demonstrated expected similarity to known seeps but also intriguing patterns, including taxa only recently discovered and of note in the evolutionary history of the world, sulfate reducing bacteria of lineages known to be associated with seepage, and Archaeal taxa which are increasingly appreciated for their role in methane cycling. The microbial community composition provides further support of the CCS being an area of active seepage as well as an enticing natural laboratory to better understand the processes and fauna associated with seepage and polar biogeochemical cycles.

**Literature Cited.**

1. [Dekas AE, Connon SA, Chadwick GL, Trembath-Reichert E, Orphan VJ. 2016 Activity and interactions of methane seep microorganisms assessed by parallel transcription and FISH-NanoSIMS analyses. *ISME J.* **10**, 678–692.](http://paperpile.com/b/drusII/3ssX9)

2. [Dumont MG, Pommerenke B, Casper P, Conrad R. 2011 DNA-, rRNA- and mRNA-based stable isotope probing of aerobic methanotrophs in lake sediment. *Environ. Microbiol.* **13**, 1153–1167.](http://paperpile.com/b/drusII/9k3s)

3. [Valentine DL. 2011 Emerging topics in marine methane biogeochemistry. *Ann. Rev. Mar. Sci.* **3**, 147–171.](http://paperpile.com/b/drusII/qZlT)

4. [Redmond MC, Valentine DL, Sessions AL. 2010 Identification of novel methane-, ethane-, and propane-oxidizing bacteria at marine hydrocarbon seeps by stable isotope probing. *Appl. Environ. Microbiol.* **76**, 6412–6422.](http://paperpile.com/b/drusII/WzuY)

5. [Bergmann GT, Bates ST, Eilers KG, Lauber CL, Caporaso JG, Walters WA, Knight R, Fierer N. 2011 The under-recognized dominance of Verrucomicrobia in soil bacterial communities. *Soil Biol. Biochem.* **43**, 1450–1455.](http://paperpile.com/b/drusII/ME05)

6. [Dunfield PF *et al.* 2007 Methane oxidation by an extremely acidophilic bacterium of the phylum Verrucomicrobia. *Nature* **450**, 879–882.](http://paperpile.com/b/drusII/TGMr)

7. [Erikstad H-A, Ceballos RM, Smestad NB, Birkeland N-K. 2019 Global Biogeographic Distribution Patterns of Thermoacidophilic Verrucomicrobia Methanotrophs Suggest Allopatric Evolution. *Front. Microbiol.* **10**, 1129.](http://paperpile.com/b/drusII/31pJ)

8. [Liu X, Li M, Castelle CJ, Probst AJ, Zhou Z, Pan J, Liu Y, Banfield JF, Gu J-D. 2018 Insights into the ecology, evolution, and metabolism of the widespread Woesearchaeotal lineages. *Microbiome* **6**, 102.](http://paperpile.com/b/drusII/sIzLV)

9. [Walker CB *et al.* 2010 Nitrosopumilus maritimus genome reveals unique mechanisms for nitrification and autotrophy in globally distributed marine crenarchaea. *Proc. Natl. Acad. Sci. U. S. A.* **107**, 8818–8823.](http://paperpile.com/b/drusII/NB8qY)

10. [Evans PN, Parks DH, Chadwick GL, Robbins SJ, Orphan VJ, Golding SD, Tyson GW. 2015 Methane metabolism in the archaeal phylum Bathyarchaeota revealed by genome-centric metagenomics. *Science* **350**, 434–438.](http://paperpile.com/b/drusII/5nLA9)

11. [Vanwonterghem I, Evans PN, Parks DH, Jensen PD, Woodcroft BJ, Hugenholtz P, Tyson GW. 2016 Methylotrophic methanogenesis discovered in the archaeal phylum Verstraetearchaeota. *Nat Microbiol* **1**, 16170.](http://paperpile.com/b/drusII/L7FL2)

12. [Spang A *et al.* 2015 Complex archaea that bridge the gap between prokaryotes and eukaryotes. *Nature* **521**, 173–179.](http://paperpile.com/b/drusII/YjGQP)


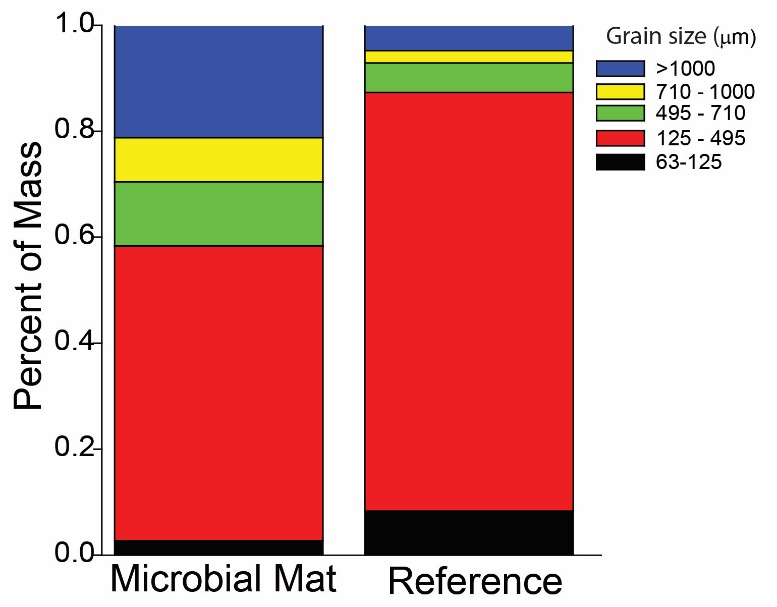


**Supplemental Figure 1**: The percent composition of grain sizes in the area of active seepage (Microbial mat) and the Reference sites at the Cinder Cones Methane Seep.


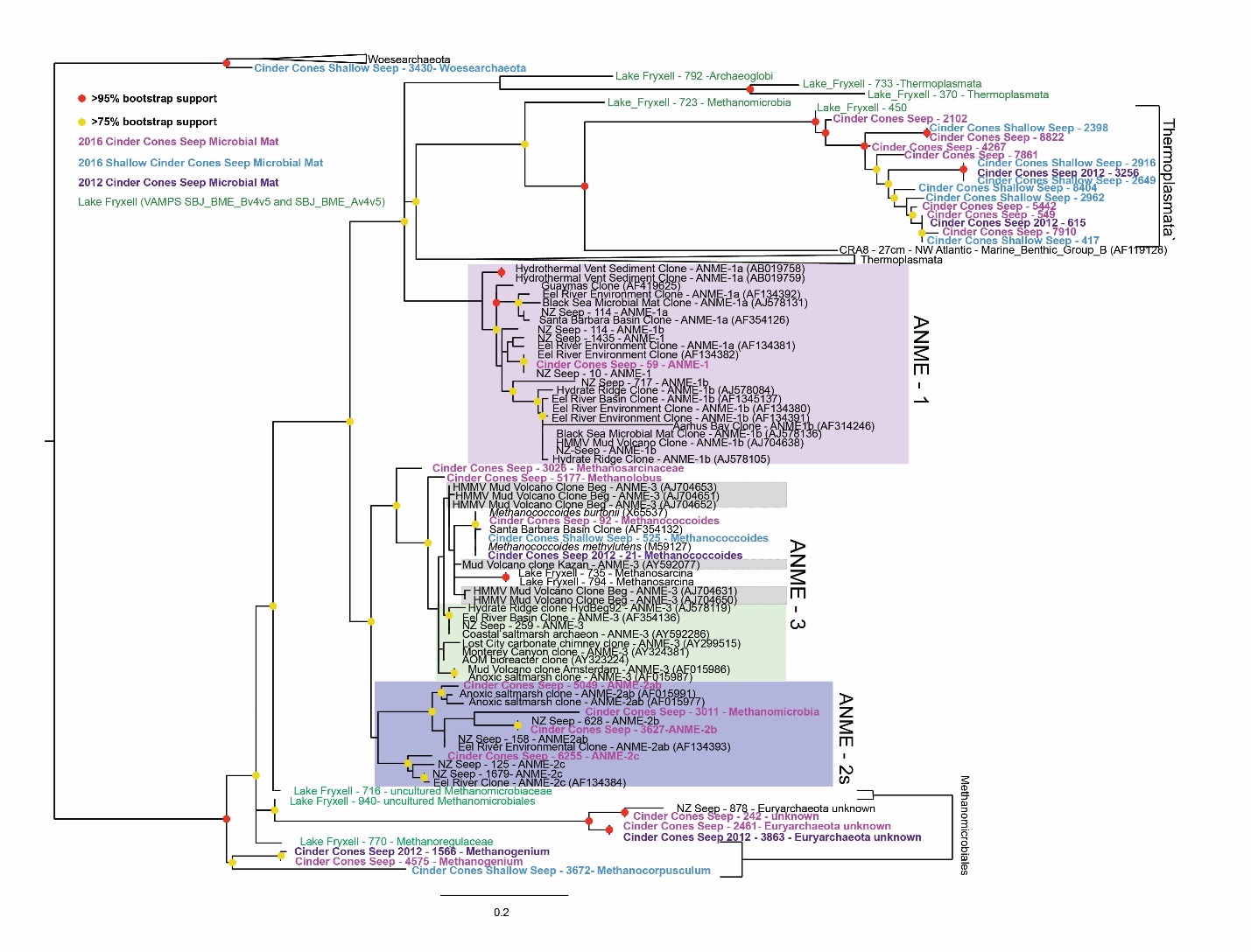


**Supplemental Figure 2:** Phylogenetic relatedness of Euryarchaeota present within the Cinder Cones Seep in comparison to other known seep taxa. Boxes indicate supported trees of particular ANME groups. The grey boxes indicate additional members of the ANME-3 group, that were separate from the main ANME-3 branch (green box).


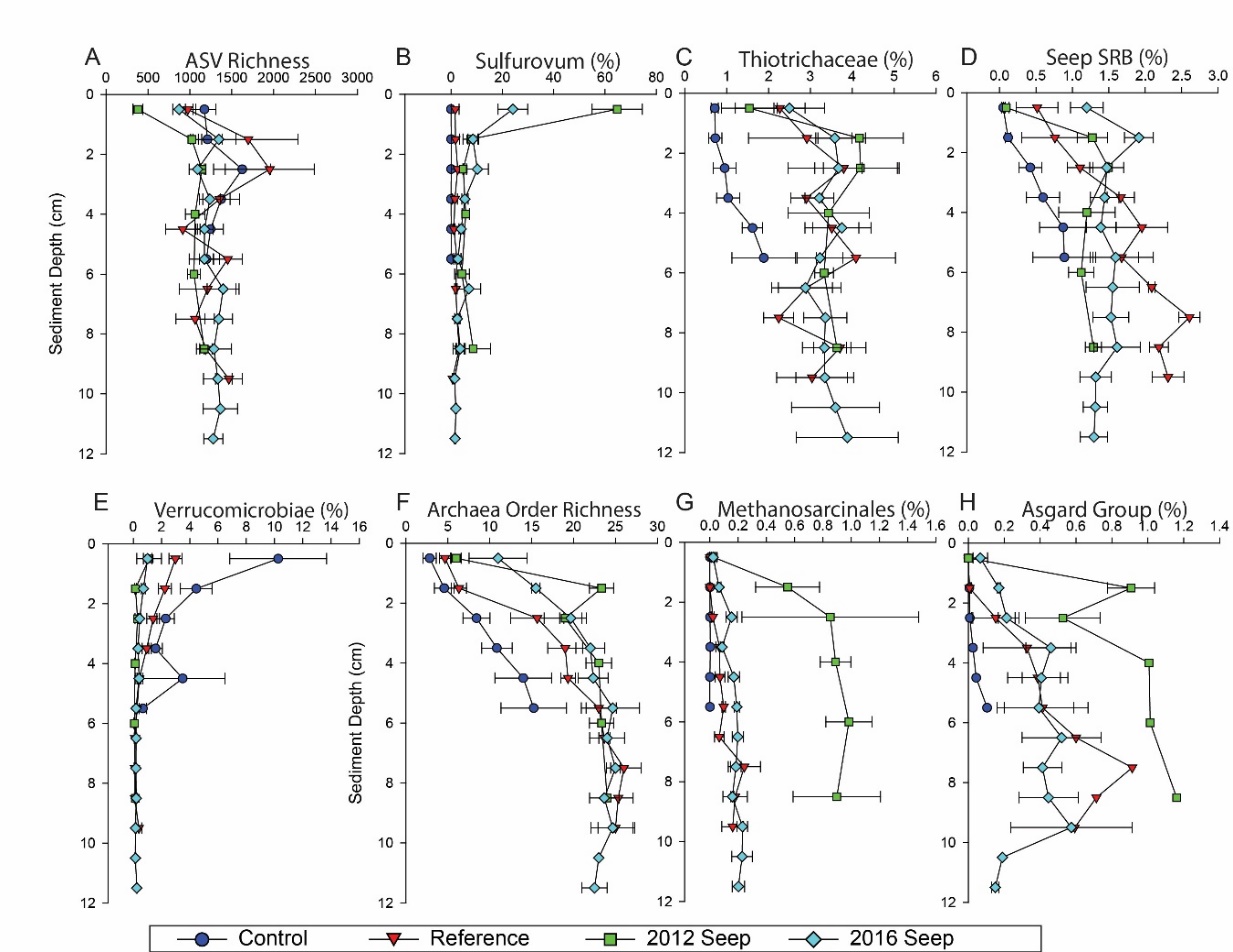


**Supplemental Figure 3:** Vertical distribution of microbial community parameters and key taxa at the Cinder Cones methane seep as inferred from relative 16S rRNA gene abundance provided as percent of the total 16S rRNA genes. A: Amplicon Sequence Variant (ASV) richness across sites and with depth. B: The percent of ASVs identified as members of the Genus *Sulforovum*, one of the most dominant members of the Campylobacterales present at the methane seeps. C: Percent of ASVs identified as Thiotrichaceae, a family that includes mat forming sulfide oxidizing bacteria. D: Percent of ASVs identified as sulfate reducing Deltaproteobacteria that were further identified as seep specific groups Seep-1, Seep-2, and Seep-4. E: Percent of ASVs identified as Verrucomicrobiae, an order that contains aerobic methanotrophs in certain environments. F: The diversity of Archaea with depth and across sites as indicated by the number of orders represented within each sample. G: Percent of ASVs identified within the Class Methanosarcinales, including taxa from the *Methanosarcina*, *Methanococcoides*, and *Methanolobus* genera. H: Percent of ASVs identified to belong to the Asgard group including Heimdallarchaeia, Lokiarchaeia, Odinarchaeia, and unclassified Asgardians. Error bars are standard error throughout and replication provided in Supplemental Table 3, note that the Jetty site was not included in the Control samples for this plot as the same depths were not sampled in both years.


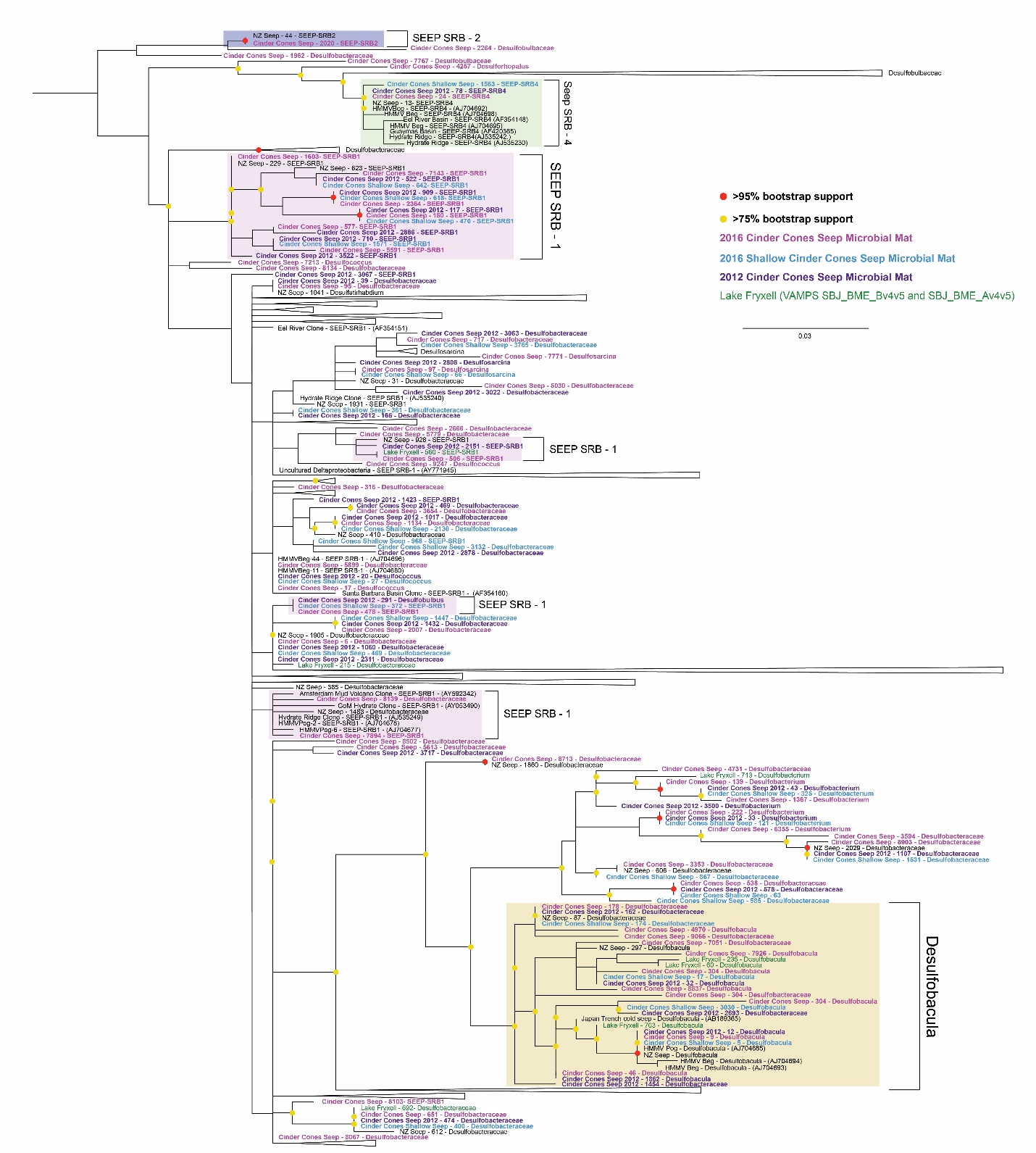


**Supplemental Figure 4:** Phylogeny of Sulfate Reducing Bacteria collected from the Cinder Cones methane seep in comparison to known seep taxa. Tree constructed using 250 bp reads. Identification is to the level of family when possible, and clades that are putatively associated with ANME, based on the literature, are indicated by colored boxes.

**Supplemental Table 1**: Vertical distribution of major ions within the sediment surface of the Cinder Cones methane seep. Variance is given by standard error. n=3


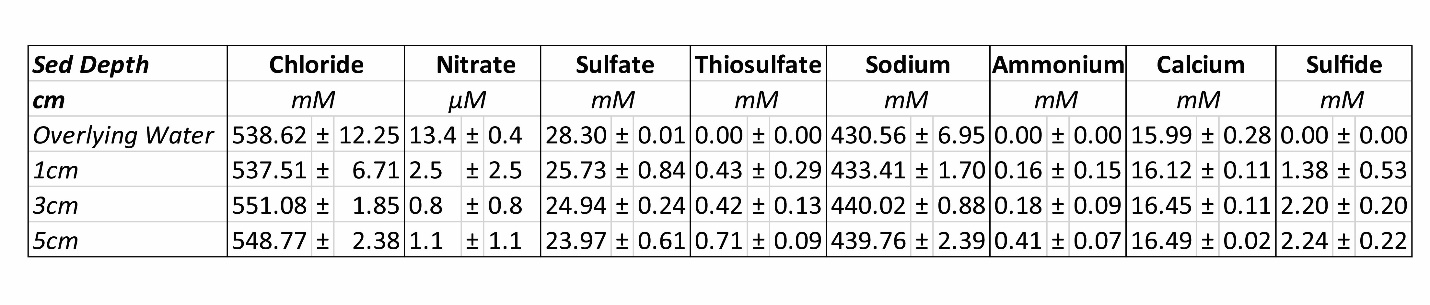


**Supplemental Table 2:** PERMANOVA results comparing samples across specific sediment depth horizons between the Cinder Cones Seep, Control and Reference Sites as visualized in Figure 3. The Shallow Seep site was not included in the PERMANOVA tests due to no replication. Replication of each depth fraction is provided in Supplemental Table 3. *Samples were pooled (mean of ASV abundance across depths) to make annual comparisons valid as samples were analyzed on 1cm depth intervals in 2016 and 1,2,or 3cm horizons in 2012.
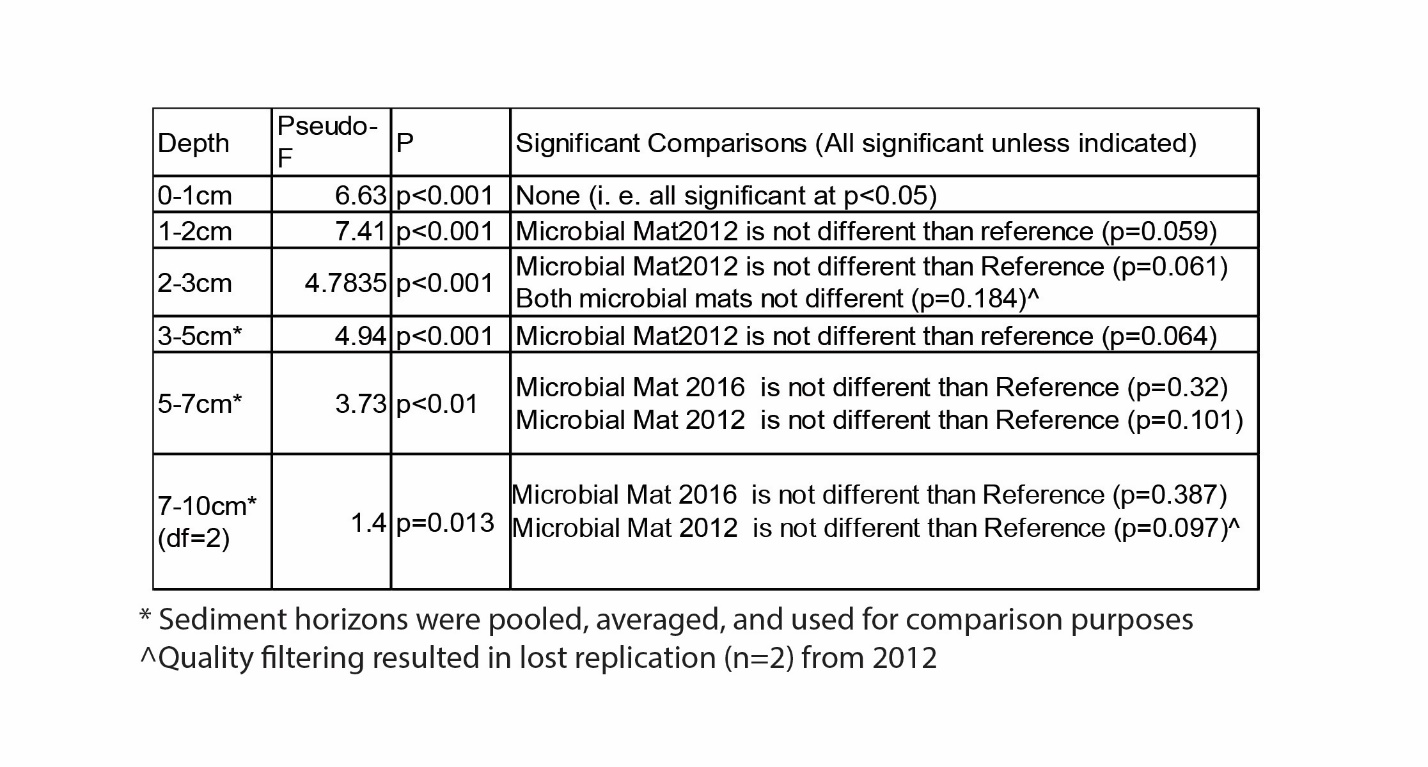


**Supplemental Table 3:** Distribution of sequencing depth and successful sampling effort. Mean sampling depth post quality control of Amplicon Sequence Variants with Standard error provided. If n=2 then variance provides the range from the mean.


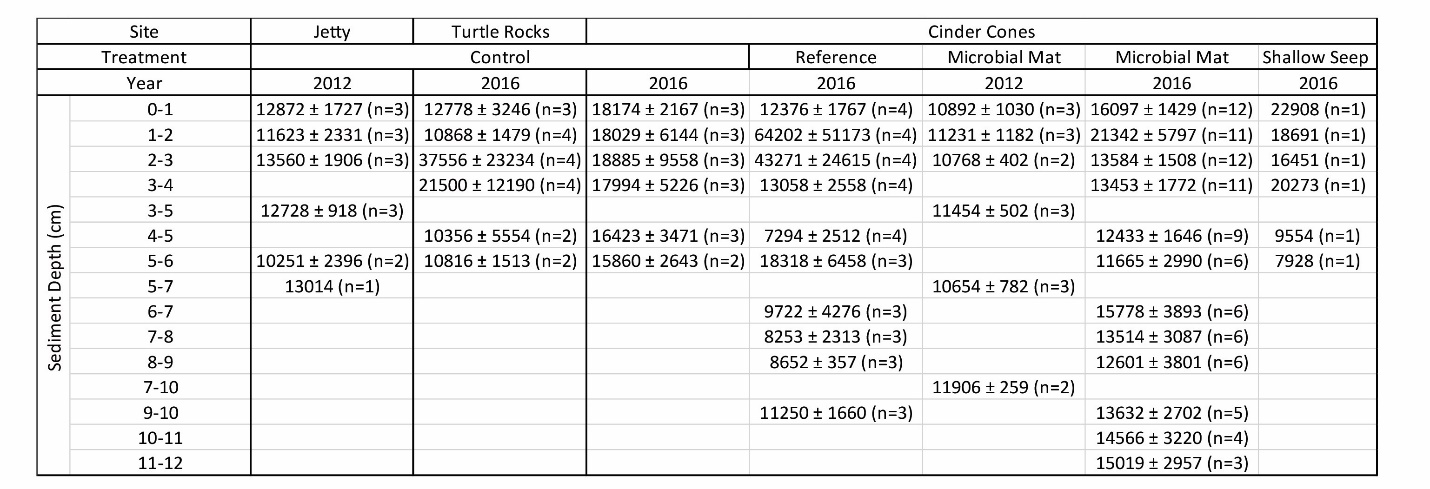


**Supplementary Data Analysis Pipeline:** Pipeline used in Qiime2 to generate Amplicon Sequence Variants.

‘Data used are available in the NCBI SRA Archive PRJNA387720

‘Resultant ASV table is provided in Dryad repository doi:10.5061/dryad.0zpc866vh

qiime tools import --type 'SampleData[SequencesWithQuality]' --input-path raw --input-format CasavaOneEightSingleLanePerSampleDirFmt --output-path demux-R1.qza

qiime quality-filter q-score \

--i-demux demux-R1.qza \

--o-filtered-sequences demux-filtered.qza \

--o-filter-stats demux-filter-stats.qza

qiime demux summarize \

--i-data demux-filtered.qza \

--o-visualization reads_summary.qzv

qiime deblur denoise-16S \

--i-demultiplexed-seqs demux-filtered.qza \

--p-trim-length 250 \

--o-representative-sequences rep-seqs-deblur.qza \

--o-table table-deblur.qza \

--p-jobs-to-start 6 \

--p-sample-stats \

--o-stats deblur-stats.qza

qiime demux summarize --i-data table-deblur.qza --o-visualization Post-Deblur.qzv

qiime metadata tabulate --m-input-file demux-filter-stats.qza --m-input-file map.tsv --o-visualization demux-filter-stats.qzv

cp rep-seqs-deblur.qza rep-seqs.qza

cp table-deblur.qza table.qza

qiime phylogeny align-to-tree-mafft-fasttree --i-sequences rep-seqs.qza --o-alignment aligned-rep-seqs.qza --o-masked-alignment masked-aligned-rep-seqs.qza --o-tree unrooted-tree.qza --o-rooted-tree rooted-tree.qza

qiime diversity core-metrics-phylogenetic --i-phylogeny rooted-tree.qza --i-table table.qza --p-sampling-depth 1103 --m-metadata-file map.tsv --output-dir core-metrics-results

qiime feature-classifier classify-sklearn --i-classifier ~/Desktop/Thurber/silva-132-99-515-806-nb-classifier.qza --i-reads rep-seqs.qza --o-classification taxonomy.qza --output-dir taxa

qiime metadata tabulate --m-input-file taxonomy.qza --o-visualization taxonomy.qzv

qiime taxa barplot --i-table table.qza --i-taxonomy taxonomy.qza --m-metadata-file map.tsv --o-visualization taxa-bar-plots.qzv

qiime taxa filter-table \

--i-table table.qza \

--i-taxonomy taxonomy.qza \

--p-include D_1__ \

--p-exclude mitochondria,chloroplast \

--o-filtered-table deblur_table_filt_contam.qza

qiime feature-table summarize \

--i-table deblur_table_filt_contam.qza \

--o-visualization deblur_table_filt_contam_summary.qzv

qiime tools export \

--input-path rarefied_table.qza \

--output-path ExportedTable

qiime tools export --input-path ../../taxonomy.qza --output-path ExportedTable

The header of the taxonomy file was modified to replace the first line to say:
#OTUID taxonomy confidence

biom add-metadata \

-i feature-table.biom \

-o feature-table_w_tax.biom \

--observation-metadata-fp taxonomy.tsv \

--sc-separated taxonomy

biom convert -i feature-table_w_tax.biom -o ASV_Feature-Table-wTax.txt --to-tsv --header-key taxonomy

In Primer 7, iterative MDS were carried out to identify a lower sequencing depth cut. This was 1900 sequence per sample and samples below this cutoff were manually removed.
